# Limited genetic diversity of N-terminal of merozoite surface protein-1 (MSP-1) in *Plasmodium ovale curtisi* and *P. ovale wallikeri* imported from Africa to China

**DOI:** 10.1186/s13071-018-3174-0

**Published:** 2018-11-16

**Authors:** Ruilin Chu, Xinxin Zhang, Sui Xu, Limei Chen, Jianxia Tang, Yuhong Li, Jing Chen, Yinghua Xuan, Guoding Zhu, Jun Cao, Yang Cheng

**Affiliations:** 1 Laboratory of Pathogen Infection and Immunity, Department of Public Health and Preventive Medicine, Wuxi School of Medicine, Jiangnan University, Wuxi, Jiangsu People’s Republic of China; 2 Key Laboratory of National Health and Family Planning Commission on Parasitic Disease Control and Prevention, Jiangsu Provincial Key Laboratory on Parasite and Vector Control Technology, Jiangsu Institute of Parasite Diseases, Wuxi, 214064 Jiangsu People’s Republic of China

**Keywords:** *Plasmodium ovale*, MSP-1, Imported malaria cases

## Abstract

**Background:**

*Plasmodium* merozoite surface protein-1 (MSP-1) is released into the bloodstream during merozoite invasion, and thus represents a crucial malarial vaccine target. Although substantial research effort has been devoted to uncovering the genetic diversity of MSP-1 for *P. falciparum* and *P. vivax*, there is minimal information available regarding the genetic profiles and structure of *P. ovale*. Therefore, the aim of the present study was to determine the extent of genetic variation among two subspecies of *P. ovale* by characterizing the MSP-1 N-terminal sequence at the nucleotide and protein levels.

**Methods:**

N-terminal of MSP-1 gene were amplified from 126 clinical samples collected from imported cases of malaria in migrant workers returning to Jiangsu Province from Africa using a conventional polymerase chain reaction (PCR) assay. The PCR products were then sequenced and analyzed using the GeneDoc, MegAlign, MEGA7 and DnaSP v.6 programs.

**Results:**

The average pairwise nucleotide diversities (*π*) of *P. ovale curtisi* and *P. ovale wallikeri* MSP-1 genes (*pomsp1*) were 0.01043 and 0.01974, respectively, and the haplotype diversity (*Hd*) were 0.746 and 0.598, respectively. Most of the nucleotide substitutions detected were non-synonymous, indicating that the genetic variations of *pomsp1* were maintained by positive diversifying selection, thereby suggesting their role as a potential target of a protective immune response. Amino acid substitutions of *P. ovale curtisi* and *P. ovale wallikeri* MSP-1 could be categorized into five and three unique amino acid variants, respectively.

**Conclusions:**

Low mutational diversity was observed in *pomsp1* from the Jiangsu Province imported malaria cases; further studies will be developed such as immunogenicity and functional analysis.

**Electronic supplementary material:**

The online version of this article (10.1186/s13071-018-3174-0) contains supplementary material, which is available to authorized users.

## Background

Malaria is one of the most serious infectious diseases of humans worldwide. An estimated 216 million cases of malaria were reported in 2016 and the global tally of malaria-caused deaths reached 445,000 [1]. Five species in the genus *Plasmodium* (*P. falciparum*, *P. vivax*, *P. malariae*, *P. ovale* and *P. knowlesi*) are known to cause human malaria under natural transmission [2]. In China, the majority of malaria cases are caused by *P. vivax* and *P. falciparum*, most of which are imported from malaria-endemic areas. Jiangsu Province, located in eastern China, was an unstable malaria transmission area and there has been no local malaria infection report since 2012. However, the number of imported malaria cases in Jiangsu ranked in the top three provinces in China, with 1799 imported malaria cases reported from 2005 to 2014 [3, 4]. As a neglected human parasite causing infection, *P. ovale* was first reported and named by Stephens in 1922 as one of the major *Plasmodium* species infecting humans [5]. *Plasmodium ovale* has a wide geographic distribution, including the Middle East, Indonesia, and Southeast Asia [6–8]. In Africa, only 0.7–10% of human malaria cases are caused by *P. ovale* infections; thus, the diagnosis of *P. ovale* is often overlooked due to the low levels of parasitemia and mixed-species malaria infections [7, 9]. Notably, approximately 300 malaria cases in China imported from Africa annually are caused by *P. ovale*. There are two subspecies of *P. ovale*, *P. ovale curtisi* (classical type) and *P. ovale wallikeri* (variant type) [10], which show dimorphism of multiple genetic loci [2].

Merozoites surface proteins (MSPs) are released into the bloodstream of the host in extracellular forms, and are thus promising vaccine targets since they play a critical role in erythrocyte invasion [11]. As the predominant member of MSPs, MSP-1 has been detected in all examined *Plasmodium* species to date, and plays an important role during erythrocyte attachment [12]. Thus, naturally acquired antibodies to MSP-1 inhibit erythrocyte invasion and are associated with protection from clinical malaria in field studies [13]. However, MSP-1-based vaccines show low protective efficacy against clinical malaria, which may be attributed to the genetic diversity of MSP-1, leading to failure of anti-malaria parasite control measures. Moreover, antigenic diversity allows the parasite to evade natural immune responses, which may cause vaccines to lose efficacy [14]. The N-terminal fragments of the MSP-1 genes of *P. falciparum* and *P. vivax* (*pfmsp1* and *pvmsp1*, respectively) show polymorphism due to selection pressure, which has hindered MSP-1-based vaccine development [15, 16]. Comparatively, the C-terminal of MSP-1 is a conserved sequence, which is carried into the infected erythrocytes during merozoite invasion [17]. A recent study demonstrated the low diversity of the *pocmsp1* and *powmsp1* gene in 10 *P. ovale* isolated from symptomatic malaria patients in Thailand, which may be related to a low transmission rate or repeated bottleneck effects [18]. However, there is limited evidence of the genetic diversity of *pomsp1*. Therefore, characterization of *pomsp1* is necessary toward understanding the population genetic structure and finding a suitable candidate for vaccine development. Accordingly, in the present study, the *pomsp1* N-terminal sequence was analyzed from the both subspecies of *P. ovale* obtained from infected migrant workers returning to China from Africa. We determined the levels of polymorphisms and nucleotide divergence of *msp1* sequences to validate the classification of *P. ovale curtisi* and *P. ovale wallikeri* as distinct species or subspecies, and trace signatures of selection.

## Methods

### Study areas and sample collection

The samples of *P. ovale curtisi* and *P. ovale wallikeri* were obtained from febrile patients at local hospitals of Jiangsu Province in China between 2012 and 2016, who had recently returned from working in tropical regions of sub-Saharan Africa endemic for malaria. A total of 126 *P. ovale-*infected blood samples were collected. Identification of the isolates was confirmed using polymerase chain reaction (PCR) of specific gene sequences. Parasite species were distinguished by PCR amplification using the real-time TaqMan PCR [3].

### PCR amplification and sequencing of the *pomsp1* N-terminal

The N-terminal nucleotide sequences of MSP-1 from *P. ovale curtisi* and *P. ovale wallikeri* were amplified by PCR using the primers designed as *pocmsp1*-Forward (5'-GAA ACG CTC GAA AAT TAT A-3') and *pocmsp1*-Reverse (5'-ACA GGA TCA GTA AAC AGA CCT T-3'), and *powmsp1*-Forward (5'-GAA ACG CTC GAA AAT TAT A-3') and *powmsp1*-Reverse (5'-ATC GGT AAA CAG ACC TTC CAT-3'), respectively. The *pocmsp1* (GenBank: KC137343) and *powmsp1* (GenBank: KC137341) sequences from the GenBank database were used as reference gene sequences. The reactions were carried out in a volume of 20 μl including 1 μl genomic DNA, 7.4 μl double-distilled water, 0.8 μl of each primer, 0.5 units DNA polymerase, and 2 mM deoxynucleoside triphosphate within 10 μl premix (2× Phanta® Max Master Mix, Nanjing, China). The PCR amplification was performed in a Mastercycler (Eppendorf, Hamburg, Germany) under the following programme: denaturation at 95 °C for 3 min; followed by 35 cycles of 95 °C for 15 s, 51 °C for 15 s and 72 °C for 30 s; and a final extension at 72 °C for 5 min. The amplified products were analyzed by 1% agarose gel electrophoresis and visualized under an ultraviolet transilluminator (Bio-Rad ChemiDoc MP, Hercules, USA). The size of the PCR products was estimated based on the mobility relative to the standard DNA marker (TRANSGEN BIOTECH, Beijing, China). PCR products were cloned into pUC57 vector and the universal primers M13F (5'-TGT AAA ACG ACG GCC AGT-3') and M13R (5'-CAG GAA ACA GCT ATG AC-3') were used for sequencing, which was performed by GENEWIZ (Suzhou, China) on an ABI 3730xl DNA Analyzer (Thermo Fisher Scientific, Waltham, USA).

### Sequence alignment and data analysis

The geographical distribution map of *P. ovale curtisi* and *P. ovale wallikeri* was constructed using Arcgis10.2 software [19]. The primary structure of the PoMSP-1 protein was predicted with a bioinformatics tool (http://smart.embl-heidelberg.de/). To evaluate the diversity of the two subspecies, the *pocmsp1* (KC137343) and *powmsp1* (KC137341) sequences were used as templates and aligned using GeneDoc2.7.0 [20]. The nucleotide sequences of *pomsp1* were translated to deduced amino acid (aa) sequences using MegAlign module of Lasergene 7 software package DNASTAR [21] and then aligned with reference aa sequences. The codon-based test of purifying selection was conducted using the MEGA7 program [22]. The rates of non-synonymous mutations (*dN*) and synonymous mutations sites (*dS*) were computed by *Z*-test using the Nei & Gojobori method [23] with the Jukes and Cantor correction and 100 bootstrap replications.

The average pairwise nucleotide diversity (*π*), haplotype diversity (*Hd*), and number of haplotypes (*H*) were calculated by DnaSP v6 [24]. The nucleotide diversity was analyzed with a window length of 50 base pairs (bp) and a step size of 3 bp using DnaSP v6. Tajima’s *D*, Fu and Li’s D*, and Fu and Li’s F* tests were used to measure the degree of deviation from neutrality [25, 26]. Phylogenetic trees of the N-terminal of MSP-1 were constructed using the neighbor-joining method according to the nucleotide sequences. The MSP-1 sequences of other *Plasmodium* species included the MSP-1 haplotypes of malaria parasites from humans (*P. falciparum*, *P. malariae*, *P. ovale* and *P. vivax*), gorillas (*P. praefalciparum*, *P. alderi* and *P. billcollinsi*), chimpanzees (*P. reichenowi*, *P. blacklocki* and *P. gaboni*), macaques (*P. knowlesi* and *P. cynomolgi*), and murine infections (*P. yoelii*, *P. chabaudi* and *P. berghei*), which were collected from the NCBI and PlasmoDB databases. Evolutionary relationships of the aligned sequences were determined using neighbor-joining approaches in MEGA 7.0.

## Results

### Geographical origin of *P. ovale curtisi* and *P. ovale wallikeri*

**Fig. 1:**
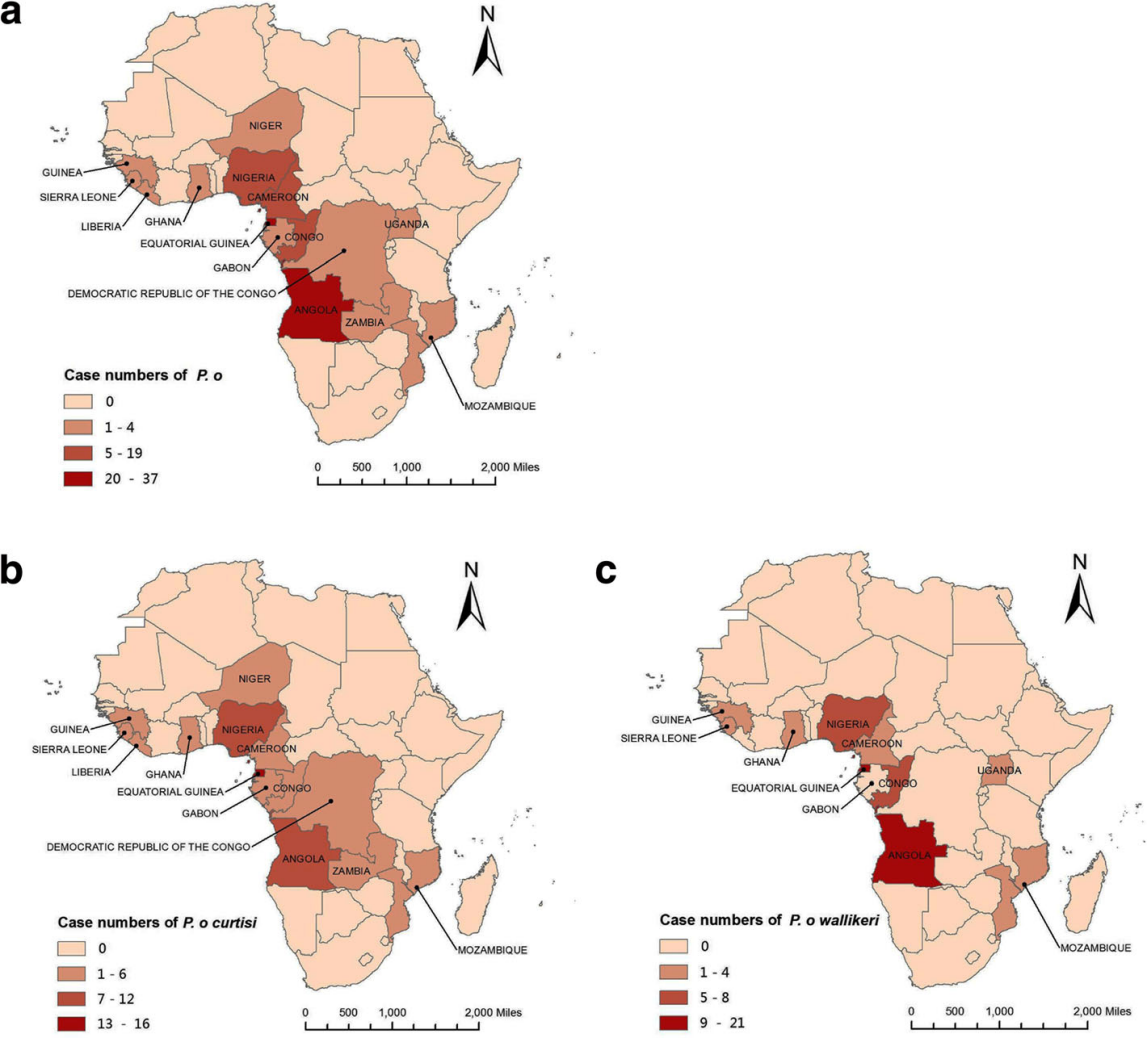
Map of Africa showing the countries of origin of *P. ovale* samples. **a** *P. ovale*. **b** *P. ovale curtisi*. **c** *P. ovale wallikeri*

The total of 126 *P. ovale* clinical isolates showed a geographical distribution across 15 countries of sub-Saharan Africa. The isolates were mainly derived from Equatorial Guinea (*n* = 37, 29.7%), Angola (*n* = 27, 21.4%), Nigeria (*n* = 19, 15.1%), and the Republic of Congo (*n* = 13, 10.3%) located on the west coast of Africa (Fig. 1). Comparatively, the *P. ovale curtisi* isolates spanned a wider range of countries (15 countries) than *P. ovale wallikeri* isolates (10 countries). Overall, 61 (48.4%) cases of *P. ovale curtisi* infection and 65 (51.6%) cases of *P. ovale wallikeri* infection were identified in this study (Table 1).

**Table 1:** Origin of imported *P. ovale curtisi* and *P. ovale wallikeri* in 2012–2016

Country	*P. ovale curtisi*		*P.ovale wallikeri*		Total
	Number	Percent	Number	Percent	Number
Angola	9	14.8	18	27.7	27
Equatorial Guinea	16	26.2	21	32.3	37
Republic of the Congo	5	8.2	8	12.3	13
Democratic Republic of the Congo	4	6.6	0	0	4
Guinea	1	1.6	2	3.1	3
Ghana	1	1.6	1	1.5	2
Gabon	1	1.6	0	0	1
Cameroon	6	9.8	4	6.2	10
Liberia	2	3.3	0	0	2
Mozambique	1	1.6	2	3.1	3
Niger	1	1.6	0	0	1
Nigeria	12	19.7	7	10.8	19
Sierra Leone	1	1.6	1	1.5	2
Zambia	1	1.6	0	0	1
Uganda	0	0	1	1.5	1
Total	61	100	65	100	126

### Characterization of PoMSP-1

**Fig. 2:**
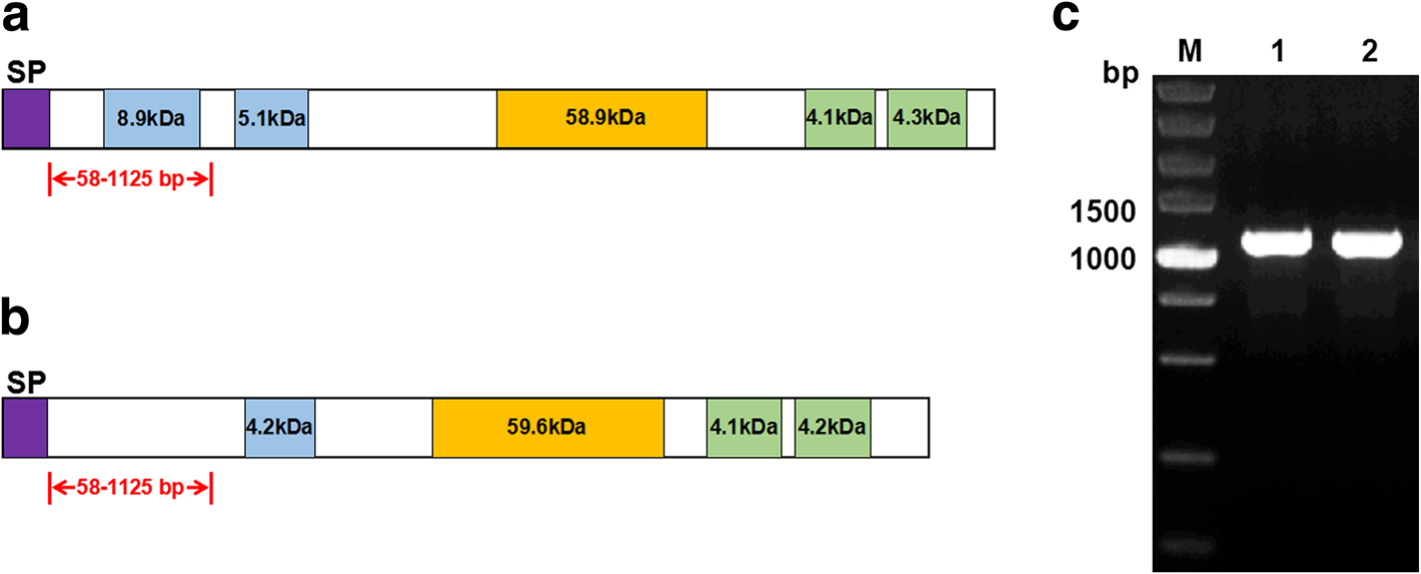
Predicted *P. ovale* MSP-1 protein primary structure and MSP-1 N-terminal fragment size. **a** *P. ovale curtisi*. **b** *P. ovale wallikeri*. **c** *pomsp1* N-terminal fragment. *Abbreviations*: M, DNA marker; 1, *pocmsp1* N-terminal fragment; 2, *powmsp1* N-terminal fragment. Purple, blue, orange and green colors indicate signal peptide, coiled-coil, Pfam and EGF domains, respectively. Arrowheads indicate *pomsp1* N- terminal for sequencing

The lengths of MSP-1 encoded by the full-length *P. ovale curtisi* (GenBank: KC137343) and *P. ovale wallikeri* (GenBank: KC137341) genes were 1727 and 1672 (aa), respectively, each beginning with a predicted 19 aa signal peptide sequence (aa 1–19). Similar to PvMSP-1 and PfMSP-1, some other specific regions were identified in the *P. ovale curtisi* predicted protein primary structure, such as a coiled-coil region (aa 288–368 and 450–495), Pfam region (aa 1011–1546), and EGF domains (aa 1624–1660 and 1667–1705) (Fig. 2a). Similarly, the PowMSP-1 predicted protein primary structure contained a signal peptide (aa 1–19), coiled-coil region (aa 458–496), Pfam region (aa 950–1491 aa), and two EGF domains (aa 1569–1605 and 1612–1650) (Fig. 2b).

### Nucleotide polymorphism of *pomsp1*

**Fig. 3:**
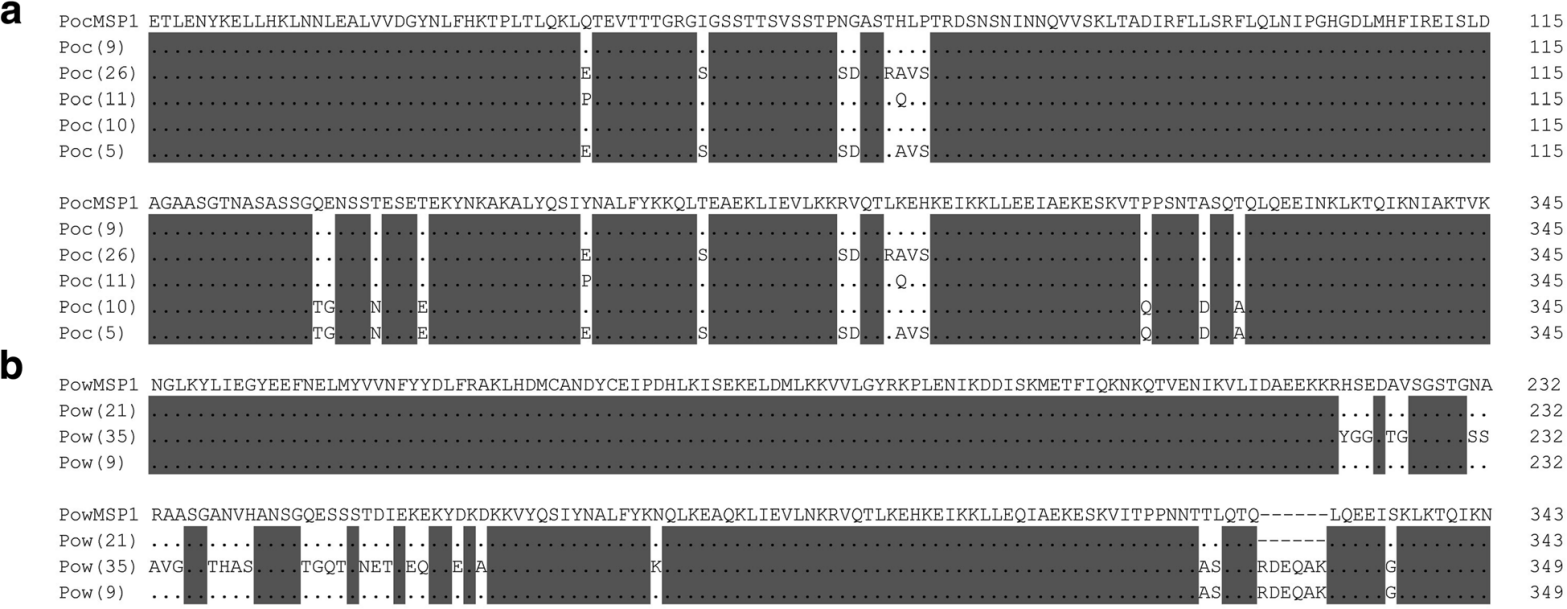
Amino acid sequence alignment of *P. ovale* MSP-1 N-terminal fragment. **a** *P. ovale curtisi*. **b** *P. ovale wallikeri*

The MSP-1 genes of the 126 *P. ovale* isolates were amplified corresponding to nucleotide positions 58–1125 (Fig. 2c). There were five genotypes of the *pocmsp1* N-terminal, and 52 isolates (85.2%) showed a non-synonymous mutation compared with the reference GH01 strain, with 44 nucleotides (67.7%) showing a non-synonymous mutation in the *powmsp1* N-terminal. Interestingly, 31 *P. ovale curtisi* isolates had 27 serine residues, while only 21 serine residues existed in the others. There were six more non-synonymous aa changes detected in *P. ovale wallikeri* isolates (Fig. 3).

**Fig. 4:**
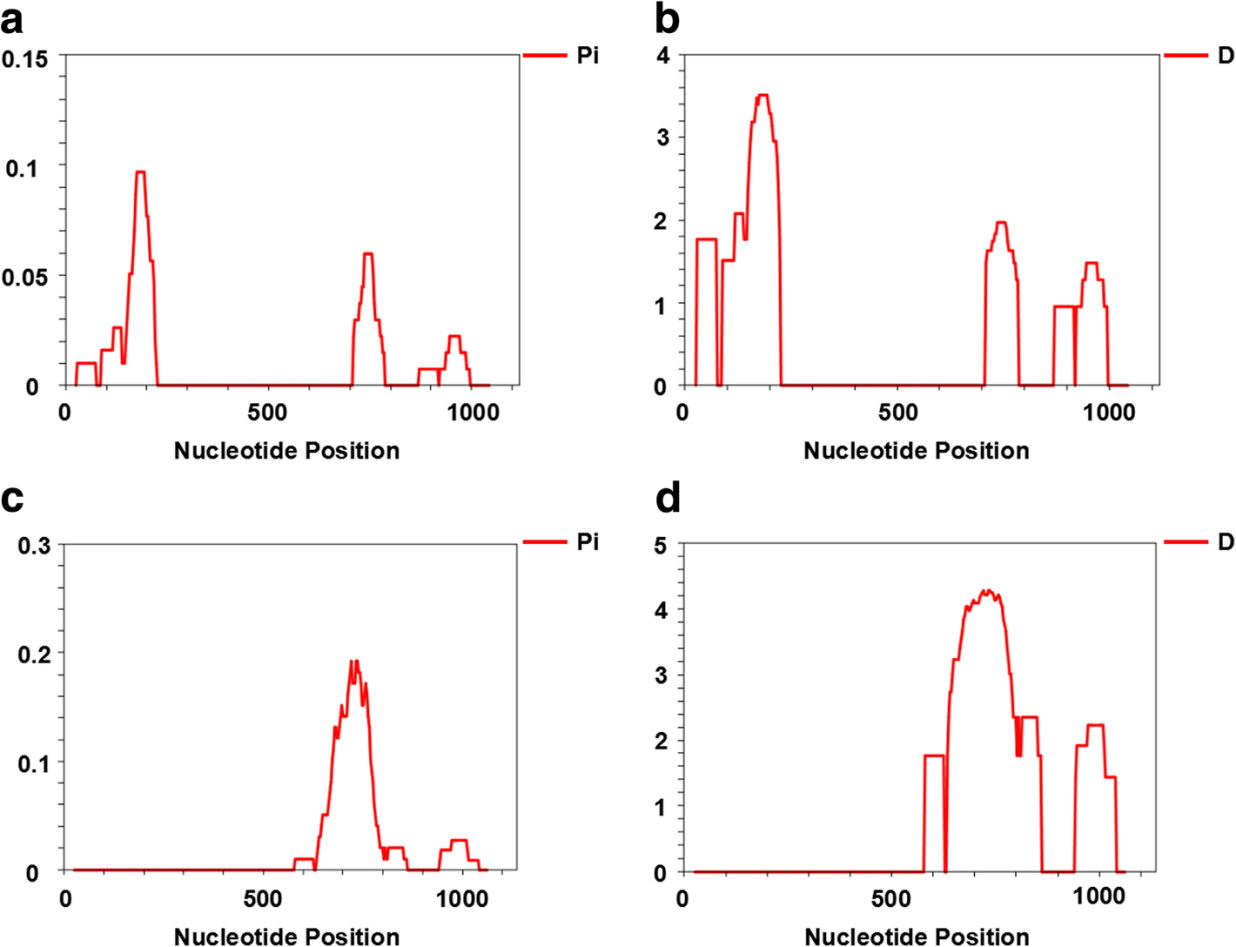
Sliding window plots of sequence diversity (*π*) and Tajima’s *D*. **a** Sequence diversity of *pocmsp1*. **b** Tajima’s *D* of *pocmsp1*. **c** Sequence diversity of *powmsp1*. **d** Tajima’s *D* of *powmsp1*

Overall, 26 single nucleotide polymorphisms (SNPs) were found among 61 samples with an average *π* value of 0.01043 in *pocmsp1*, and 42 SNPs were detected among 65 samples with an average *π* value of 0.01974 in *powmsp1*. A sliding method plot with a window length of 50 bp and a step size of 3 bp using DnaSP v6 revealed a *π* value in the range of 0–0.09688 and 0–0.19221 for *pocmsp1* and *powmsp1*, respectively. The conserved region was observed from 0.2–0.7 kb length in *pocmsp1* and before 0.6 kb in *powmsp1* with approximate *π* values of 0 (Fig. 4). The haplotype (gene) diversity of *pocmsp1* could be categorized into five distinct haplotypes with an estimated *Hd* of 0.746 and three distinct haplotypes with an estimated *Hd* of 0.598 in *powmsp1* samples (Table 2). The average number of nucleotide differences (*k*) for *pocmsp1* and *powmsp1* was 11.139 and 21.081, respectively.

**Table 2:** Estimates of nucleotide diversity, natural selection, haplotype diversity and neutrality indices of *pomsp1* N-terminal fragment

Type	No. samples	G + C content (%)	No. haplotypes	*Hd*	Diversity ± SD		Tajima’s *D*	Fu & Li’s D*	Fu & Li’ s F*
					Nucleotide	Haplotype			
*P. ovale curtisi*	61	34.1	5	0.746	0.01043 ± 0.00061	0.746 ± 0.035	3.22138	1.81498	2.77112
*P. ovale wallikeri*	65	35.2	3	0.598	0.01974 ± 0.00055	0.598 ± 0.036	4.57287	2.00379	3.5575

### Genetic population structure based on the *pomsp1* N-terminal

The population genetic structure of the *P. ovale* isolates was analyzed based on the MSP-1 N-terminal gene polymorphisms applied to the codon-based test of purifying selection according to average *dS* and *dN* values within each isolate. There was clear evidence of positive selection or diversifying selection in *P. ovale* MSP-1 [Prob = 1.000, *dS* - *dN* = -0.06 (*pocmsp1*), -0.478 (*powmsp1*)]. In addition, Tajima’s *D*, Fu and Li’s D* and F* tests rejected a neutral model of polymorphism occurrence with values for *pocmsp1* (Tajima’s *D* = 3.22138, *P* < 0.01; Fu and Li’s D* = 1.88498, *P* < 0.02; Fu and Li’s F* = 2.77112, *P* < 0.02) and *powmsp1* (Tajima’s *D* = 4.57287, *P* < 0.001; Fu and Li’s D* = 2.00379, *P* < 0.02; Fu and Li’s F* = 3.5575, *P* < 0.02), respectively (Table 2).

### Phylogenetic analysis

**Fig. 5:**
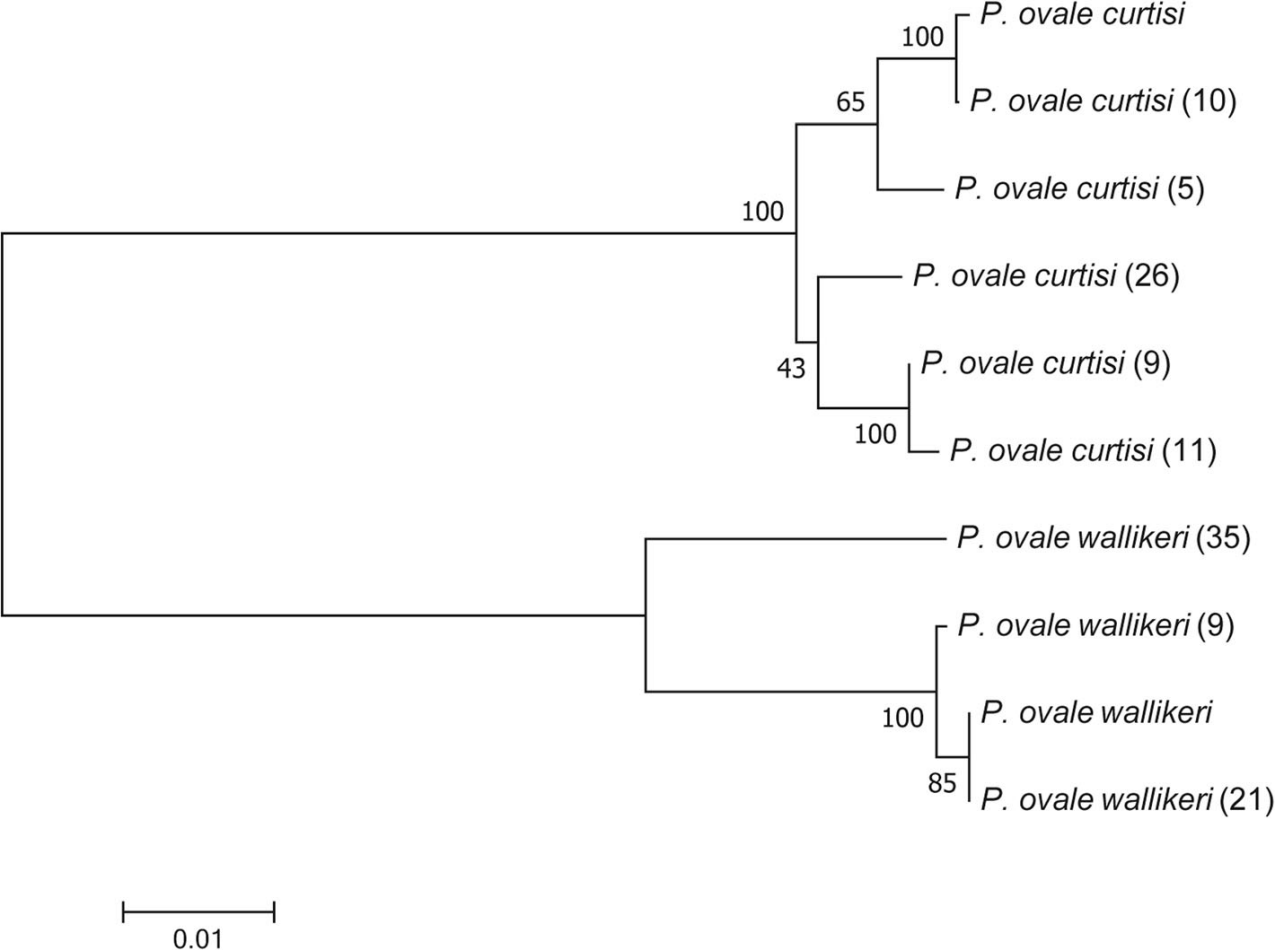
Phylogenetic relationship of MSP-1 N-terminal genes within *pomsp1* sequences based on neighbor-joining method

As predicted based on the low level of genetic diversity and signature of positive selection described above, a close phylogenetic relationship was detected in *pomsp1* sequences between the subspecies based the branch lengths of *pocmsp1* and *powmsp1* with 100% bootstrap support (Fig. 5). Phylogenetic trees of 26 MSP-1 gene alleles from the 18 species of *Plasmodium* in human and non-human primates were constructed using the neighbor-joining method (Additional file 1: Figure S1).

## Discussion

The life-cycle of the malaria parasite alternates between the human host and the mosquito vector, which is complex with extensive genetic and antigenic diversity across different stages of the parasite’s life [14]. The genetic diversity of *P. ovale* might have impacted malaria transmission and the success of malaria control strategies. Gaining a deeper understanding of the mechanisms and patterns of genetic recombination and sequence variation may help in designing a vaccine that could represent the worldwide repertoire of polymorphic malaria surface antigens [27]. The sequences of *pocmsp1* and *powmsp1* showed a low level of diversity in a limited number of Asian isolates [18]. Hence, we analyzed the N-terminus of *pocmsp1* (61 isolates, 48.4%) and *powmsp1* (65, 51.6%), and found that *pocmsp1* was more conserved than *powmsp1* with 26 (14 synonymous, 12 non-synonymous) and 42 (26 synonymous, 16 non-synonymous) sites of nucleotide diversity, respectively.

Neutrality tests were further performed to determine the signatures of natural selection on the MSP-1 N-terminal fragment of *P. ovale*. Significantly positive values for these statistics reflect an excess of intermediate frequency alleles, which can result from population bottlenecks or balancing selection. The sequences for subspecies *P. ovale curtisi* and *P. ovale wallikeri* were further divided into five and three branches that were all within the same evolutionary branches. The MSP-1 N-terminal sequences placed the two subspecies in a distinct bifurcating branch, and the split of *pocmsp1* and *powmsp1* seems to be relatively more recent. Therefore, the MSP-1 N-terminal sequences of *P. ovale curtisi* and *P. ovale wallikeri* support the ancient divergence times of the malaria parasite lineage [28].

The *Z*-test (*dS* - *dN* < 0) indicated that strong positive or purifying selection within the parasite population. These results were in agreement with previous studies which suggested that such mechanisms might be in favour of parasites to evade targeted host immune responses [29]. In addition, the genetic diversities at the *P. ovale* MSP-1 N-terminal [*π* = 0.01043 ± SD 0.00061 (*pocmsp1*), *π* = 0.01974 ± SD 0.00055 (*powmsp1*)] were lower compared to that of *P. falciparum* and *P. vivax* [30], which may be related to the lower transmission rate of *P. ovale* from diverse geographical origins [8]. These findings were similar to previously published data demonstrating a low level of sequence diversity of the MSP-1 gene in *P. ovale* [18].

The intragenic recombination of MSP-1 gene is a major informative pattern at the level of population sequence diversity. The frequency of allelic recombination has important guiding significance for the population structure of parasites [31]. A previous study demonstrated that *P. falciparum* has a low level of genetic diversity in areas with low transmission rates and high level of sequence diversity in areas with high transmission rates [32]. High mutational diversity was observed in *pvmsp1* isolated from Thailand northwestern region [33]. The level of nucleotide diversity in both *pocmsp1* and *powmsp1* N-terminal sequences detected in this study showed lower magnitude than that reported for *pvmsp1* and *pfmsp1* [34, 35].

## Conclusions

This study provides valuable reference information on the genetic diversity of *P. ovale curtisi* and *P. ovale wallikeri* isolates imported from Africa to China based on analysis of the MSP-1 N-terminal sequence. To our knowledge, this is the first report of the genetic diversity, selection signature, and population structure of the N-terminal of *pomsp1* gene from an African population. The low level of genetic diversity indicated that these genes are under purifying selection. Therefore, these sequences have potential for vaccine development, which requires further investigation of the immunogenicity and antigenicity of *P. ovale* MSP-1.

## Additional file

Additional file 1: **Figure S1.** Neighbor-joining tree of 26 unique alleles of the gene encoding *msp1* from 18 *Plasmodium* parasite species. (TIF 3756 kb)

## Abbreviations

MSP-1: Merozoite surface protein-1
PCR: Polymerase chain reaction
aa: Amino acid
*Hd*: Haplotype diversity
SNP: Single nucleotide polymorphism
*dN*: Rates of non-synonymous substitutions
*dS*: Rates of synonymous substitutions
H: Haplotype
*Hd*: Haplotype diversity
*k*: Average number of pairwise nucleotide differences within the population
*π*: Nucleotide diversity
