# Supplementary figures and images for: Limited genetic diversity of N-terminal of merozoite surface protein-1 (MSP-1) in Plasmodium ovale curtisi and P. ovale wallikeri imported from Africa to China
Source: Parasit Vectors. 2018 Nov 16;11:596. doi: 10.1186/s13071-018-3174-0 (PMC6240192; doi:10.1186/s13071-018-3174-0)

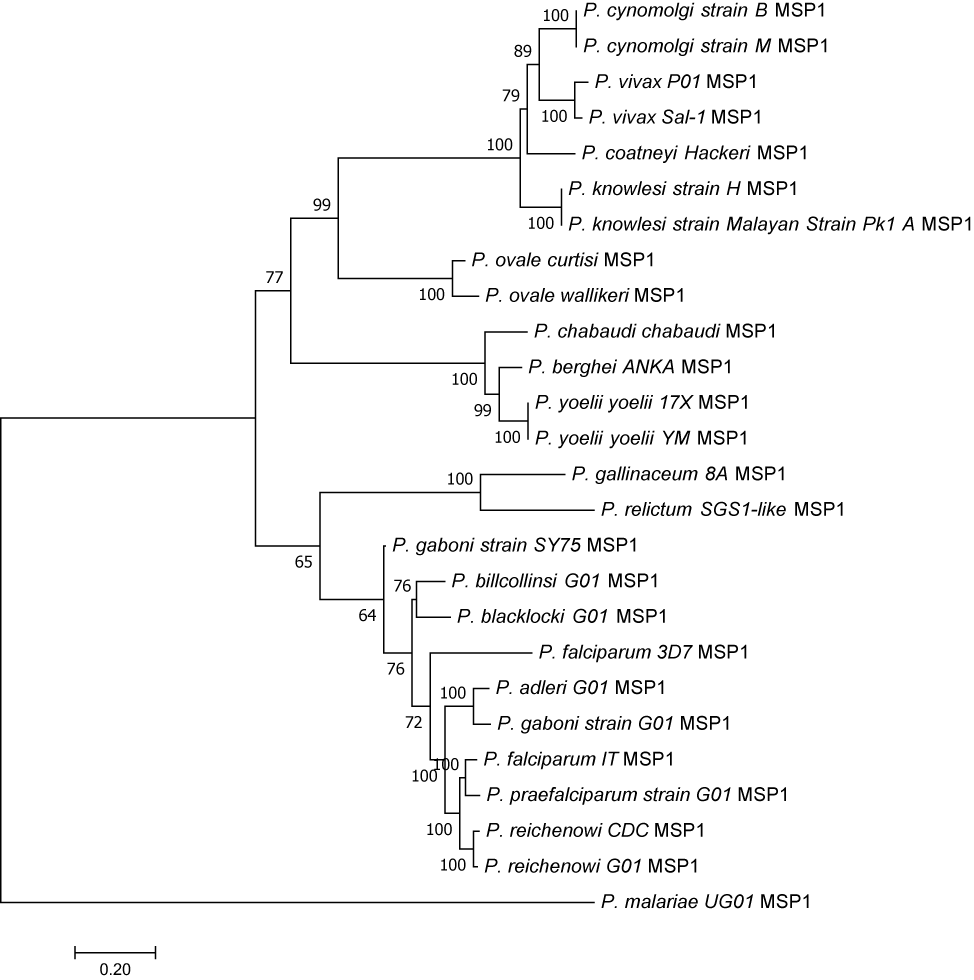

Supplement: Supplementary file 1 — Figure S1. Neighbor-joining tree of 26 unique alleles of the gene encoding msp1 from 18 Plasmodium parasite species. (TIF 3756 kb) [file 13071_2018_3174_MOESM1_ESM.tif]
